# Temperature driving a mass killer: assessing the risk of trematode outbreaks for New Zealand seabirds

**DOI:** 10.1017/S0031182026101796

**Published:** 2026-04

**Authors:** Jerusha Bennett, Mikey Little, Jane Kitson, Robert Lewis, Sally Carson, Jake Edwards-Ingle, Robert Poulin

**Affiliations:** 1 Department of Zoology, University of Otago, Dunedin, New Zealand; 2 Hokonui Rūnanga, Southland, New Zealand; 3 Tohu Environmental, Southland, New Zealand; 4 Department of Marine Science, University of Otago, Dunedin, New Zealand

**Keywords:** *Copiatestes*, euphausiid, parasite, plankton, seabird anklets

## Abstract

Global warming is widely recognized as a key driver of current and future changes in marine ecosystems. Parasitic trematodes are highly sensitive to temperature changes, which can lead to drastic impacts on surrounding communities. *Copiatestes* spp. (Family Syncoelidae) are relatively little-known trematodes with atypical life cycles that have been associated with a mass mortality event of seabirds in the Chatham Islands. As they forage at sea, seabirds get their legs tangled with the sticky, free-living infective stages of *Copiatestes*, which impairs their ability to take off and land. We tested the impact of seasonally fluctuating sea temperature on the dynamics of *Copiatestes thyrsitae* at various life stages (infecting second-intermediate host, *Nyctiphanes australis* euphausiid, and the third free-living infective stage in the water column) by sampling plankton biweekly for 12 months in Otago Harbour, New Zealand. We reveal that higher temperatures are significantly correlated with increases in prevalence and abundance of *Copiatestes* infections in euphausiids, with a rapid response observed within days to weeks following temperature increases. No correlation was observed between temperature and abundance of free-living stages. Infected euphausiids were smaller in size compared to uninfected individuals. The higher infection levels in euphausiids following warmer temperatures suggest a heightened risk of entanglement for coastal seabirds at these times. Smaller-bodied, surface-feeding seabird species that consume euphausiids are particularly at risk of entanglement. Our findings suggest that even short-term heatwaves can lead to higher risk of seabirds being entangled with *Copiatestes* filaments, with potentially dire ecological consequences during mass parasite releases.

## Graphical abstract

**Figure:**
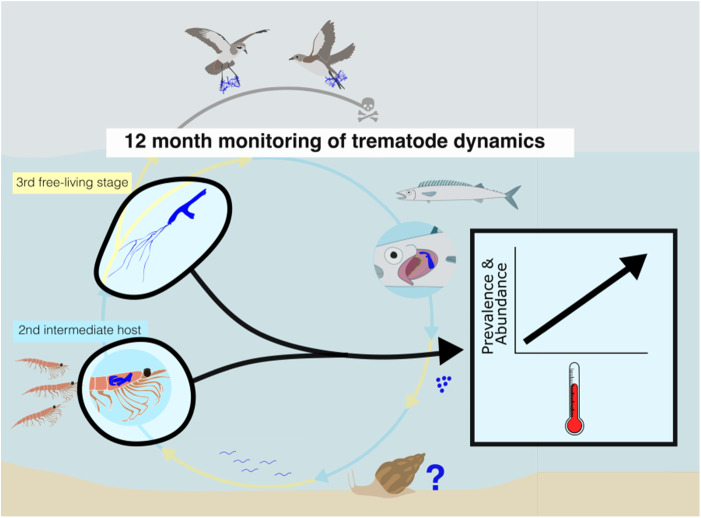

## Introduction

Climate change is widely recognized as the greatest threat to the diversity, stability and functioning of the world’s marine ecosystems (Brierley and Kingsford, 2009; Hoegh-Guldberg and Bruno, 2010; Doney et al., 2012). Seabirds are among the many organisms likely to be severely impacted by climate change (Dias et al., 2019). They play key roles in marine ecosystems, from nutrient transfer to habitat connectivity (Signa et al., 2021), and they are widely seen as indicators of ecosystem health (Piatt and Sydeman, 2007; Parsons et al., 2008). A recent overview of the impact of heatwaves (and global warming in general) on seabirds covers several mechanisms ranging from habitat modification to physiological stress (Piatt et al., 2024). Surprisingly, there is not a single mention in this review of the possible impact of rising sea temperatures on the dynamics, severity and geographic distribution of parasites and diseases affecting seabirds. This is a glaring omission, as global warming has been widely recognized for over two decades as a key driver of current and future changes in parasite infections across marine habitats (Marcogliese, 2001; Harvell et al., 2002; Poulin and Mouritsen, 2006; Burge et al., 2014; Byers, 2020).

In coastal ecosystems where the life cycles of helminth parasites have been well resolved, such as that of southern New Zealand (Bennett et al., 2023), seabirds have been shown to play key roles as definitive hosts. Among marine parasites, including those infecting seabirds, that are most likely to be impacted by global warming, trematodes stand out. Several stages of their life cycle are notoriously sensitive to temperature. In particular, small changes in water temperature can have huge effects on the rate at which infective stages, i.e. cercariae, are produced within and released from molluscan first intermediate host (Poulin, 2006; Selbach and Poulin, 2020) as well as their survival and infectivity to the second intermediate host (Koprivnikar, 2010; Morley and Lewis, 2015). These temperature-driven effects on trematode biology can have drastic consequences on host populations (Mouritsen and Jensen, 1997; Mouritsen et al., 2005) and communities (Mouritsen et al., 2018). Although these impacts are often documented for invertebrate intermediate hosts, there is no reason they cannot extend to vertebrates as well.

**Figure 1.**
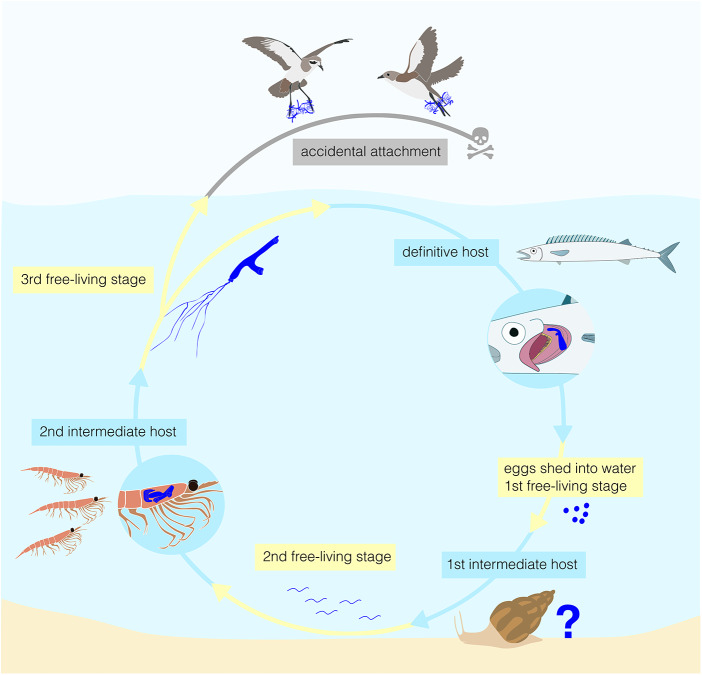
Schematic illustrating the presumed life cycle of *Copiatestes thyrsitae.* The second intermediate host and subsequent free-living stage are the focus of this study. Figure 1 long description.

The present study investigates the seasonal response of an unusual trematode, *Copiatestes thyrsitae* Cowcroft, 1948, to changes in its thermal environment, and the potentially serious consequences of any rise in its abundance for seabirds. *Copiatestes* spp. (Family Syncoelidae) are relatively little known, enigmatic parasites with atypical life cycles and large impacts on their surrounding communities. Presumably, they follow a three-host life cycle (Gibson and Bray, 1977), including a molluscan first intermediate host (although none have been discovered yet), a euphausiid second intermediate host (Morales-Ávila et al., 2015; Bennett et al., 2023) and a fish definitive host (Manter, 1954; Gibson and Bray, 1977; Bennett et al., 2023) (Figure 1). While most other trematode taxa have only two free-living stages, the miracidium stage that seeks out the first intermediate host, and the cercaria stage, between first and second intermediate hosts (Poulin, 2007), *Copiatestes* spp. have an additional one between the second and definitive hosts, as noted by several authors (Odhner, 1911; Dollfus, 1966; Claugher, 1976; Bennett et al., 2023) (Figure 1). Within this stage, individuals possess a pair of bladder-like bodies and byssus-like filaments extending from the terminal tip of the body (Gibson, 1976). The purpose of this additional life stage and morphological structures remains elusive, although Claugher (1976) hypothesized that diurnal migrations of euphausiids may have promoted *Copiatestes* spp. to evolve the use of a third free-living stage, with the sticky filaments likely increasing the probability of coming into physical contact with their fish definitive hosts (Claugher, 1976). The existence of this additional stage has ecological consequences for marine life, as non-target organisms can become entangled in *Copiatestes*’ sticky filaments (e.g. Claugher (1976).

Reports of *Copiatestes* entanglement with seabird tarsi highlight their ecological importance. These entanglements, often referred to as ‘seabird anklets’, have been documented in various species of petrels and prions (Family Procellariidae), particularly around off-shore islands in the South Atlantic, Antarctic and South Pacific (Claugher, 1976; Furness, 1984; Imber, 1984; Ryan, 1986). Of the few times this association has been documented in the literature, impacts on seabird movement, is often negligible. However, one record exists of *Copiatestes* being associated with the mass mortality of an estimated over 200 000 seabirds in the Chatham Islands in the 1970s (Claugher, 1976). Birds presumably starved because they were unable to take off from land after being entangled with anklets while feeding at sea. Six bird species were involved in that event; since then, and not during any mortality event, Imber (1984) reported anklets present (but not limiting tarsi movement) on three of these native petrels and prions. A large-scale survey off the coast of South Africa also revealed that anklets are associated with smaller bodied species and those with a diet including euphausiids (Imber, 1984).

Here, we present the results of a 12-month monitoring program that aimed to assess the occurrence of *Copiatestes thyrsitae* in its second intermediate host, *Nyctiphanes australis* G. O. Sars, 1883 (hereafter referred to as euphausiid) and that of its third free-living stages in Otago Harbour, South Island, New Zealand. We specifically test the causal role of sea temperature in driving temporal changes in the abundance of this parasite, and thus in the risks of entanglement for seabirds. Our findings reveal how the abundance of this parasite peaks at higher temperatures, and that it is nonetheless present throughout the year in both its second intermediate host and as free-living stages. Finally, we comment on the bird species at highest risk of *Copiatestes* entanglement during any future heatwave events.

## Materials and methods

**Figure 2.**
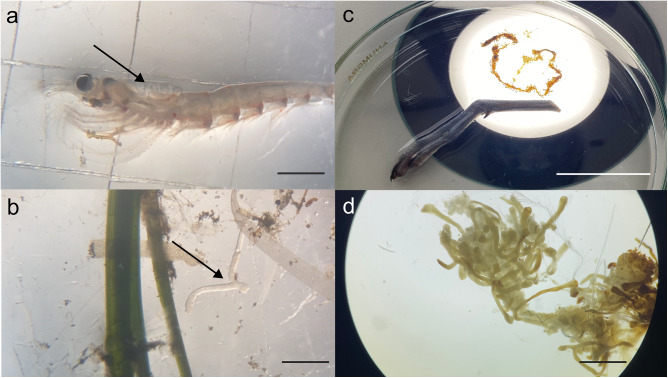
Photo micrographs of (a) euphausiid (*Nyctiphanes australis*) infected with *Copiatestes thyrsitae*, (b) *C. thyrsitae* third free-living stage, attached to surrounding material, (c) numerous *Copiatestes* individuals and filaments removed from a bird tarsus, and, (d) a close up of *C. thyrsitae* individuals entangled from a bird tarsus. Scale a–b, *d* = 2 mm, *c* = 2.5 cm. Figure 2 long description.

This study was conducted between September 2023 and August 2024 at the New Zealand Marine Studies Centre, University of Otago in Dunedin, New Zealand. Approximately every 2 weeks, one 250-micron (48 cm diameter) plankton net was deployed at the end of the centre’s wharf (approximate GPS coordinates: latitude −45.827925, longitude 170.639939) and left overnight for at least 16 h. Owing to the hydrology of the Otago Harbour, there can be two distinct biodiversity signals depending on whether sampling occurs in the upper or lower harbour (one being benthic and the other oceanic). The sample location is placed advantageously in that it receives both outgoing tidal water and incoming coastal water bodies with each tidal cycle allowing for representation of species from both. Each plankton sample was either processed immediately after collection, or kept in a 30 L bucket with an air bubbler for a few hours until processing. This was to reduce the possibility of euphausiid death and incorrect identification of free-living stages of *C. thyrsitae* that would emerge after host death. In each sample, the following was recorded: the total number of second intermediate hosts of *C. thyrsitae* (euphausiid *N. australis*), whether each euphausiid was infected (Figure 2a) and if so by how many *C. thyrsitae*, and the number of *C. thyrsitae* in their third free-living stage (Figure 2b). Infections and the free-living stage was identified with the use of a dissecting microscope whereby the whole plankton sample was screened and life stages identified (see Figure 2b for example of third free-living stage). The third free-living stage was counted if a *Copiatestes* individual was in plankton sample, but not infecting a host. If a deceased krill host was in the sample with a broken carapace (meaning it is feasible that the *Copiatestes* had just emerged because their host had died) then this was not counted as free-living. We also measured the body length of a subsample of infected and uninfected euphausiids under a dissecting microscope: total body length was measured from the tip of the rostrum to the end of the tail fan.

For each plankton collection, we calculated various parasite descriptors following Bush et al. (1997). These included prevalence defined as the percent of hosts infected with *C. thyrsitae* within each sample, mean intensity defined as the average number of *C. thyrsitae* individuals per infected euphausiid host, and mean abundance defined as the average number of *C. thyrsitae* individuals per euphausiid host (including infected and uninfected ones). Sea surface temperature at the location of collection was recorded as a single daily reading by Portobello Marine Laboratory as part of an existing long-term time series of single daily recordings (Shears and Bowen, 2017).

For a graphical representation of temporal changes in prevalence over the study duration, we plotted prevalence with Clopper–Pearson 95% confidence intervals for each sample, the number of free-living stages recovered per sample, and the sea surface temperature at the time of sampling.

All data analyses and visualizations were conducted in R (R Core Team, 2023) using packages stats (R Core Team, 2023) and ggplot2 (Wickham et al., 2025). Generalized linear models (GLMs) were used to test the influence of temperature on parasite parameters (including prevalence, mean abundance, mean intensity and number of free-living stage individuals found as response variables) with function glm(). The data for all parasite descriptors was non-normal (quantified with Shapiro–Wilk tests [Shapiro and Wilk, 1965]) and all but number of free-living stage individuals were over-dispersed (dispersal values greater than 1 [Gelman and Hill, 2006]). Therefore, distribution and link functions for the GLMs were allocated as follows: prevalence = quasi-binomial with logit function, mean abundance and mean intensity = quasi-poisson with log function and number of free-living stage individuals (count data) = poisson with log function. For each GLM, various temperature profiles were used as our explanatory variables to account for any potential time lag between peaks in temperature and increased shedding of cercariae by the unknown first intermediate host, resulting in euphausiid infections. These temperature profiles were as follows: Day 0 (temperature on the day of plankton collection), Week 1 (average temperature for the 0–7 days preceding sampling), Week 2 (average temperature for the 8–14 days preceding sampling), Week 3 (average temperature for the 15–21 days preceding sampling), Month 1 (average temperature for the 0–30 days preceding sampling), Month 2 (average temperature for the 31–60 days preceding sampling), Month 3 (average temperature for the 61–90 days preceding sampling), and Max Month 1 (maximum sea surface temperature observed during the month prior to sampling). We used deviance explained (a measure of model fit – the proportion of variance in response variable accounted for by the model) to determine which time lag provided the best fit for each parasite parameter. To account for testing multiple comparisons and increased risk of Type 1 error, we applied a Bonferroni correction to our resulting *p*-values (Haynes, 2013).

Finally, to determine whether infected euphausiids had smaller body lengths on average than uninfected individuals, we compared the two groups with a Mann–Whitney test.

## Results

**Figure 3.**
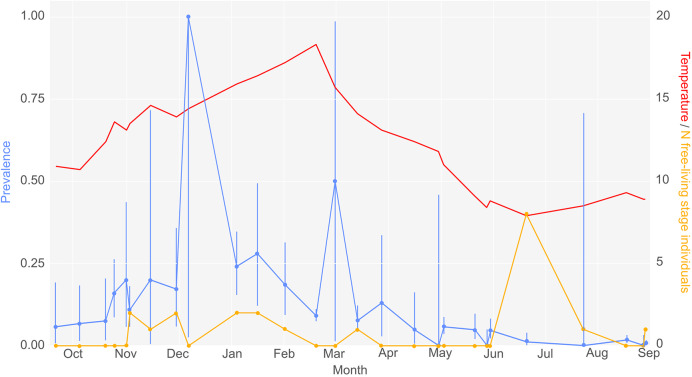
Prevalence of infection with Clopper–Pearson 95% confidence intervals, number of free-living stage individuals recovered from plankton net, and daily sea surface temperature per plankton sampling event of *Copiatestes* in euphausiid (*Nyctiphanes australis*) at Otago Harbour, New Zealand between September 2023 and August 2024. See Table 1 for sample sizes. Figure 3 long description.

A total of 3954 euphausiids were collected from Otago Harbour between September 2023 and August 2024 (Table 1). Of these, 237 individuals were infected with *Copiatestes thyrsitae*, harbouring a total of 303 parasites, with an overall prevalence of 5.9% across all sampling dates. The number of hosts collected per sample ranged from 1 to 973, with an average of 146 euphausiids per sample. The prevalence of *C. thyrsitae* ranged from 0 to 100% (average prevalence = 13.5%). Most infections involved just one *C. thyrsitae* individual per host, although the largest sample (*n* = 973) included several multiple infections (34 double, 1 triple, and 1 quadruple). We recovered 26 free-living stages of *C. thyrsitae* from the water column within plankton nets during our collections. These free-living stages were found in about 40% of sampling dates. Overall, prevalence, mean abundance, intensity, number of free-living stages recovered, and sea surface temperature varied substantially across the sampling period of this study (Table 1; Figure 3).

**Table 1.** Parasite data from plankton nets deployed repeatedly in Otago Harbour, Dunedin, New Zealand between September 2023 and August 2024, including number (*N*) of euphausiids (*N. australis*) obtained, number infected with *Copiatestes thyrsitae*, number of free-living *C. thyrsitae* individuals obtained, prevalence, mean abundance, and mean intensity for infected euphausiids Table 1 long description.

Sample date	*N* euphausiids	*N* infected	*N* free-living stages	Prevalence (%)	Mean abundance	Mean intensity
21 Sep 2023	35	2	0	5.7	0.09	1.5
5 Oct 2023	45	3	0	6.7	0.11	1.67
20 Oct 2023	40	3	0	7.5	0.10	1.33
25 Oct 2023	75	12	0	16	0.17	1.08
1 Nov 2023	20	4	0	20	0.20	1
3 Nov 2023	111	12	2	10.8	0.14	1.26
15 Nov 2023	5	1	1	20	0.20	1
30 Nov 2023	29	5	2	17.2	0.17	1
7 Dec 2023	1	1	0	100	–	1
4 Jan 2024	83	20	2	24.1	0.28	1.15
16 Jan 2024	25	7	2	28	0.36	1.29
1 Feb 2024	54	10	1	18.5	0.24	1.3
19 Feb 2024	973	89	0	9.2	0.13	1.46
1 Mar 2024	2	1	0	50	0.50	1
14 Mar 2024	198	15	1	7.6	0.09	1.2
28 Mar 2024	25	3	0	12	0.13	1
16 Apr 2024	42	2	0	4.8	0.05	1
30 Apr 2024	6	0	0	0	–	–
3 May 2024	348	17	0	4.9	0.06	1
21 May 2024	145	7	0	4.8	0.05	1
28 May 2024	73	0	0	0	–	–
30 May 2024	219	10	0	4.6	0.06	1.2
20 Jun 2024	181	2	8	1.1	0.01	1
23 Jul 2024	3	0	1	0	–	–
17 Aug 2024	579	8	0	1.4	0.02	1.2
27 Aug 2024	113	0	0	0	–	–
28 Aug 2024	524	3	1	0.6	0.01	1

**Figure 4.**
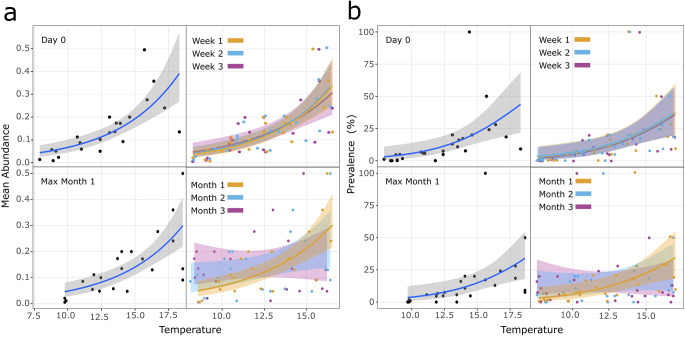
Relationships between temperature and (a) mean abundance and (b) prevalence (%) of *Copiatestes* infecting euphausiids (*Nyctiphanes australis*) in Otago, New Zealand. Each panel displays the relationship between different temperature profiles (Day 0, Max Month 1, Week 1, Week 2, Week 3, Month 1, Month 2 and Month 3) and parasite parameter based on generalized linear models. Lines represent statistically significant relationships, and shaded areas denote 95% confidence intervals. Figure 4 long description.

Significant correlations were observed between parasite infection parameters and temperature across time periods prior to host collection (Table 2; Figure 4). Strong significant positive relationships were observed between mean abundance and prevalence, and sea surface temperature (Table 2). As temperature increased, abundance of *C. thyrsitae* within infected hosts and percentage of infected hosts increased (Table 2; Figure 4). This relationship was observed for temperature combinations of up to 1 month prior to collection of the hosts, including temperature on the collection day (Day 0), the average temperature of 1 (Week 1), 2 (Week 2), and 3 (Week 3) weeks prior to collection, average temperature for 1 month prior (Month 1), and the highest maximum temperature observed for 1 month prior to collection (Max Month 1) (Table 2). The strength and significance of these positive correlations were relatively consistent across time profiles (deviance explained ranged 32–64% and *p*-values ≤ 0.05). The average temperature 2 weeks prior to sampling (Week 2) explained the highest deviance compared to other time profiles for predicting mean abundance and prevalence (deviance explained = 64% and 59%, respectively). The coefficient estimates suggested that with every 1 °C increase in the average temperature 2 weeks prior to sampling, the log-odds of prevalence and abundance increase is 0.24 and 0.25, respectively. No infection parameter (prevalence, mean abundance, mean intensity, number of free-living stages) was significantly correlated with the average temperature of the sea surface 2  or 3 months prior to collection of hosts (Month 2 and Month 3; Table 2), except for prevalence at Month 2 which was slightly significant (*p* = 0.01) with moderate fit (Table 2). We found no effect of sea surface temperature and the number on free-living individuals and mean intensity for any of the time profiles tested (Table 2).

**Table 2.** Generalized linear models exploring relationships between parasite descriptors (prevalence, mean abundance, mean intensity and number (*n*) of free-living individuals) and sea surface temperature taken over different time periods prior to sampling, including: temperature of water on collection day (Day 0), average temperature of 1, 2, and 3 weeks and months prior to collection (Week 1, Week 2, Week 3, Month 1, Month 2, Month 3), and the maximum temperature of the previous month (Max Month 1). *P*-values reported as uncorrected and Bonferroni corrected in brackets. SE denotes standard error. Statistically significant results are bolded Table 2 long description.

	Parasite descriptors			
	Prevalence (Quassi-binomial, logit link)			
	*t*-value	*p*-value (corrected)	Coefficient estimate (SE)	Deviance explained
**Day 0**	**4.27**	**<0.001 (<0.01)**	**0.17 (0.04)**	**48.15%**
**Week 1**	**4.7**	**<0.001 (<0.001)**	**0.20 (0.04)**	**54.03%**
**Week 2**	**4.93**	**<0.001 (<0.001)**	**0.24 (0.05)**	**59.27%**
**Week 3**	**4.06**	**<0.001 (<0.01)**	**0.21 (0.05)**	**49.52%**
**Month 1**	**4.12**	**<0.001 (<0.01)**	**0.22 (0.05)**	**49.28%**
Month 2	2.78	0.01 (0.08)	0.19 (0.06)	32.83%
Month 3	1.39	0.17 (1.0)	0.10 (0.07)	9.45%
**Max Month 1**	**4.31**	**<0.001 (<0.01)**	**0.20 (0.05)**	**51.30%**
	Mean abundance (Quassi-Poisson, log link)			
	*t*-value	*p*-value (corrected)	Coefficient estimate (SE)	Deviance explained
**Day 0**	**5.18**	**<0.001 (<0.001)**	**0.22 (0.05)**	**58.48%**
**Week 1**	**5.59**	**<0.001 (<0.001)**	**0.24 (0.04)**	**62.95%**
**Week 2**	**5.77**	**<0.001 (<0.001)**	**0.25 (0.04)**	**64.92%**
**Week 3**	**4.26**	**<0.001 (<0.01)**	**0.21 (0.05)**	**48.64%**
**Month 1**	**4.67**	**<0.001 (<0.01)**	**0.23 (0.05)**	**53.15%**
Month 2	2.09	0.05 (0.39)	0.12 (0.06)	17.52%
Month 3	0.38	0.71 (1.0)	0.02 (0.06)	0.76%
**Max Month 1**	**4.98**	**<0.001 (<0.001)**	**0.22 (0.04)**	**55.37%**
	Mean intensity (Quassi-Poisson, log link)			
	*t*-value	*p*-value (corrected)	Coefficient estimate (SE)	Deviance explained
Day 0	0.25	0.52 (1.0)	0.08 (0.01)	2.10%
Week 1	0.20	0.84 (1.0)	<0.01(0.01)	0.20%
Week 2	0.14	0.89 (1.0)	<0.01 (0.01)	0.09%
Week 3	0.14	0.89 (1.0)	<0.01 (0.01)	0.10%
Month 1	<0.01	0.99 (1.0)	<0.01 (0.01)	<0.01%
Month 2	−1.01	0.32 (1.0)	−0.01 (0.01)	4.82%
Month 3	−1.11	0.28 (1.0)	−0.01 (0.01)	5.72%
Max Month 1	−0.01	0.99 (1.0)	<−0.01 (0.01)	<0.01%
	Number of third stage free-living individuals (Poisson, log link)			
	*z*-value	*p*-value (corrected)	Coefficient estimate (SE)	Deviance explained
Day 0	−0.96	0.34 (1.0)	−0.07 (0.08)	0.02%
Week 1	−0.43	0.67 (1.0)	−0.03 (0.08)	<0.01%
Week 2	−0.26	0.80 (1.0)	−0.02 (0.08)	<0.01%
Week 3	−0.81	0.42 (1.0)	−0.07 (0.08)	0.01%
Month 1	−0.86	0.39 (1.0)	−0.08 (0.09)	0.01%
Month 2	−0.60	0.55 (1.0)	−0.05 (0.08)	<0.01%
Month 3	−0.16	0.87 (1.0)	−0.01 (0.07)	<0.01%
Max Month 1	−0.96	0.34 (1.0)	−0.08 (0.08)	0.02%

**Figure 5.**
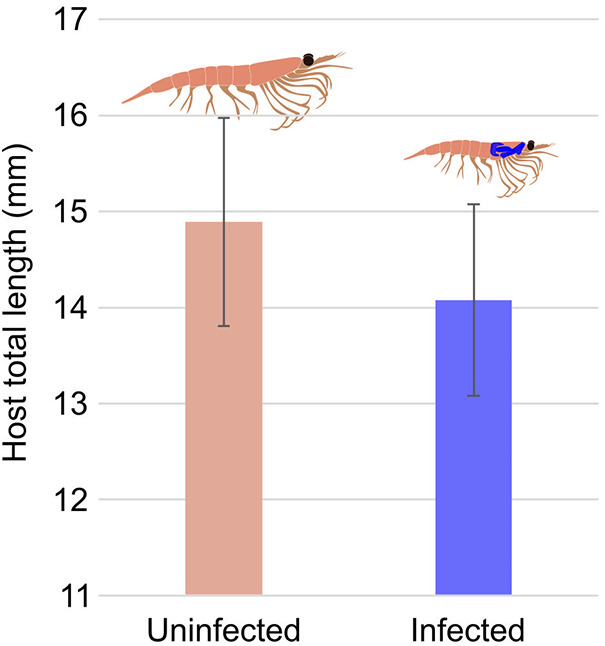
Average size (mm) of euphausiid (*Nyctiphanes australis*) uninfected and infected by *Copiatestes thyrsitae* in Otago Harbour, New Zealand. *N* uninfected = 100, *N* infected = 11. Error bars represent 95% confidence intervals. Figure 5 long description.

We found a statistically significant difference between the average total length of infected euphausiids and that of uninfected euphausiids (*p* = 0.019, *w* = 354.5; Figure 5). The median size of uninfected euphausiids was 7.14% greater than infected euphausiids (median size: uninfected = 14 mm, infected = 15 mm).

## Discussion

Rising sea temperatures resulting from global warming are predicted to interact with parasitism to impact marine life (Marcogliese, 2001; Harvell et al., 2002; Poulin and Mouritsen, 2006; Burge et al., 2014; Byers, 2020). However, these impacts may not necessarily involve disease, instead increases in parasite abundance can affect marine organisms in indirect ways, even organisms that are not part of a parasite’s life cycle. Here, we demonstrate that over a full year, periods of warmer temperatures are immediately followed by greater prevalence and abundance of infections by the trematode *Copiatestes thyrsitae* in its euphausiid hosts. The period between high temperatures and higher infection levels is from days to a few weeks, suggesting a rather rapid response of the parasite and an acceleration of this part of its life cycle as a function of higher temperatures. We found no such relationship between temperature and the number of third free-living stages, i.e. the stage that poses risks for seabirds. However, given the low numbers of free-living stages caught in our plankton net, such relationships are difficult to detect. Nevertheless, greater infections of euphausiids will invariably lead to the release of more free-living stages. Therefore, our results suggest that even short-term heatwaves can lead to high risks of seabirds becoming entangled with the *C. thyrsitae* filaments, with potentially dire consequences (see Claugher, 1976).

Until recently, *Copiatestes* spp. had only been reported infecting central to outer shelf pelagic fish species as definitive hosts in New Zealand, namely kahawai *Arripis trutta*, snapper *Pagrus auratus*, blue and silver warehou *Seriolella brama* and *S. punctata*, skipjack tuna *Katsuwonus pelamis*, jack and Murphy’s mackerel *Trachurus novaezelandiae* and *T. murphy*, and barracouta *Thyrsites atun* (Hine et al., 2000). If *Copiatestes* was not present in shallow coastal environments, then the risk of entanglement in coastal bird species would be negligible. However, Bennett et al. (2023) recovered adult *Copiatestes* infecting small schooling coastal fish, i.e. mullet *Aldrichetta forsteri* and sprat *Sprattus antipodum*, meaning this parasite’s distribution extends to inshore areas. While our plankton net set-up filtered water from both incoming and outgoing tides, it is all ultimately neritic inshore water, further supporting the idea that *Copiatestes* poses a potential risk for coastal species beyond those in order Procellariiformes that have previously been implicated in filament entanglement.

The risk of *Copiatestes* entanglement will vary across bird species depending on body size, foraging ecology and behaviour (Imber, 1984). Seabirds observed with anklets are typically small-bodied, consume euphausiids and/or exhibit feeding behaviours whereby their legs touch the water surface for extended periods of time (Ryan, 1986) (see anklet example Figure 2c–d). Smaller bodied birds may not have the physical strength to break up filaments once entangled (which are sometimes up to 60 mm long; Imber, 1984), meaning they may be at higher risk of entanglement in future.

The majority of seabirds identified with *Copiatestes* anklets are small-bodied species that also consume krill and exhibit hydroplaning feeding behaviour (Ryan, 1986). This includes fairy prion (*Pachyptila turtur*, Tītī Wainui), broad-billed prion (*Pachyptila vittata*, Pararā), and the white-faced storm petrel (*Pelagodroma marina*, Takahikare-moana) (Claugher, 1976; Furness, 1984; Imber, 1984; Ryan, 1986). These birds all exhibit feeding strategies that involve surface foraging and flying close to the water surface during which they strike the water surface with their feet (Hernandez and Arroyo, 2023). The white-faced storm petrel was the highest impacted species in the *Copiatestes-*associated mass mortality event in the Chatham Islands in 1970s, and the most frequently observed with anklets from a subsequent bird anklet survey in 1986 (Claugher, 1976; Ryan, 1986). Commonly referred to as the ‘Jesus Christ bird’ due to its apparent ability to walk on water while feeding (Southey, 2022), this behaviour likely increases its exposure to free-living trematode stages.

An exception among small seabirds associated with anklets is the common diving petrel (*Pelecanoides* urinatrix), a pursuit diver. These birds use pattering behaviour to launch from feeding areas, propelling themselves in flight for distances up to 50 m (Ryan and Nel, 1999). Not only would this behaviour likely increase entanglement risk, but the fact that they seek out and predominantly consume *Nyctiphanes australis* as part of their diet in New Zealand and Australian waters (Fromant et al., 2021) also contributes to their entanglement risk. While *Copiatestes* are present in coastal inshore ecosystems, there is no evidence of entanglements for inter- and subtidal bird species, like cormorants, oystercatchers and gulls. To date, only species from the order Procellariiformes are associated with trematode anklets. Procellariiformes are one of the most globally threatened avian groups (Croxall et al., 2012), and in future, we can expect that for time periods up to one month post higher temperature spikes, bird species most at risk should be monitored for potential entanglement.

We found evidence that infected euphausiids are smaller in size than uninfected ones. This could be explained by either (1) if smaller euphausiids due to differences in behaviour are more likely to encounter free-living larval stages (shed from their first intermediate hosts) than larger euphausiids, or (2) this parasite is detrimental to the growth of their hosts. Considering the large size of *Copiatestes* within the host haemocoel, we assume that the second explanation is more likely. Larval parasite size is a trade-off between the benefits of being larger and the cost that a larger size imposes on their transmission success due to the negative impacts on host survival (Parker et al., 2003). *Copiatestes* are not dependent on their euphausiid hosts’ survival for successful transmission to their fish definitive host (instead having a third free-living stage), meaning they may attain larger than typical sizes without the constraints that many other parasites experience.

How do increases in temperature lead to an increase of *Copiatestes* within the coastal ecosystem? The increase in presence of *Copiatestes* within euphausiids is most likely due to greater parasite production within the first intermediate host. Unfortunately, no first intermediate hosts have been identified for either of the two known *Copiatestes* species, worldwide. Based on the fact that most other trematodes use various molluscs as first intermediate hosts, we assume that the first intermediate host in this case is also a mollusc (Gibson et al., 2002). Warmer temperatures accelerate the asexual production of cercariae within the first intermediate hosts and trigger their release into the water column (Poulin, 2006). Monitoring outbreaks using larval stages as indicators may be a viable way to monitor this, and other problematic parasites in marine ecosystems, especially when it is not appropriate or ethical to directly monitor the animals that are being impacted.

## Data Availability

Samples of *Copiatestes* from the haemocoel of krill, *Nyctiphanes australis* are accessioned at Te Papa museum, under accession W.003620.

## Figure 1 Long description

The diagram illustrates the life cycle of Copiatestes thyrsitae, featuring a three-host cycle. It begins with eggs shed into water, leading to the first free-living stage. The first intermediate host is a mollusk, indicated by a question mark. The cycle progresses to the second free-living stage, followed by the second intermediate host, a euphausiid. The third free-living stage is depicted with bladder-like bodies and filaments. The definitive host is a fish, shown with a diagram of its internal structure. Arrows indicate the progression through each stage. Accidental attachment to seabirds is shown, highlighting potential ecological consequences. Each stage and host is labeled, with arrows indicating the direction of the cycle.

Navigate back to Figure 1.

## Figure 2 Long description

The image A showing a translucent, elongated specimen oriented left to right on a pale background. A black arrow points to a structure near the upper side of the specimen. A black scale bar sits near the lower right. The image B showing a pale background with a thick, vertical, dark green strand near the left side. A thin, curved, translucent strand lies to the right of the green strand. A black arrow points toward the thin curved strand. A black scale bar sits near the lower right. The image C showing a circular field with a dark outer ring and a bright center. A thin, translucent strand enters from the lower left toward the center. A brown, irregular looped strand lies near the center. A black scale bar sits near the lower right. The image D showing a circular field with a dark outer ring and a pale center. A clustered, branching, pale green mass occupies the right half of the field, with multiple thin extensions projecting leftward. A darker brown clump sits at the far right edge.

Navigate back to Figure 2.

## Figure 3 Long description

The graph displays three datasets over months from October to September. The x-axis is labeled Month, with ticks for Oct, Nov, Dec, Jan, Feb, Mar, Apr, May, Jun, Jul, Aug, Sep. The left y-axis is labeled Prevalence, ranging from 0.00 to 1.00. The right y-axis is labeled Free-living (No. of individuals), ranging from 0 to 20. The prevalence is shown as a blue line, temperature as a red line and free-living individuals as orange bars. Key trends include a peak in prevalence around December, a smaller spike in March and a notable free-living peak in July. Temperature shows a gradual decline from December to June, with minor fluctuations thereafter. The graph highlights seasonal variations in prevalence and free-living counts, with temperature changes influencing these patterns.

Navigate back to Figure 3.

## Figure 4 Long description

The figure is divided into two main labeled sections, a and b. Section a plots mean abundance on the vertical axis, ranging from 0.0 to 0.5, against temperature on the horizontal axis. Section b plots prevalence in percent on the vertical axis, ranging from 0 to 100 percent, against temperature on the horizontal axis. In both sections, temperature is measured in degrees and each section contains two subplots: one for Day 0 and one for Max Month 1. Within each subplot, multiple series are drawn representing Week 1, Week 2, Week 3, Month 1, Month 2 and Month 3, differentiated by distinct line styles and point markers. Shaded bands around each fitted line represent 95 percent confidence intervals. Fitted lines are present only where relationships are statistically significant. Section a, subplot Day 0: The horizontal axis spans approximately 7.5 to 17.5 degrees. The vertical axis spans 0.0 to 0.5. Scatter points are present throughout, with a fitted line rising from approximately 0.05 at 10 degrees to approximately 0.4 at 17 degrees. The series for Week 1, Week 2 and Week 3 show upward-sloping fitted lines across the same temperature range. Week 2 and Week 3 series show steeper slopes compared to Week 1. Several scatter points fall above 0.4 near the upper temperature range, representing potential outliers. Section a, subplot Max Month 1: The horizontal axis spans approximately 10 to 17.5 degrees. The vertical axis spans 0.0 to 0.5. Series for Month 1, Month 2 and Month 3 are plotted. Month 1 shows an upward-sloping fitted line rising from approximately 0.05 at 10 degrees to approximately 0.35 at 17 degrees. Month 2 and Month 3 series show wider confidence intervals and less steep slopes, with Month 3 showing the widest shaded band. Section b, subplot Day 0: The horizontal axis spans approximately 7.5 to 17.5 degrees. The vertical axis spans 0 to 100 percent. A fitted line rises from approximately 10 percent at 10 degrees to approximately 75 percent at 17 degrees. Week 1, Week 2 and Week 3 series follow similar upward trajectories. Week 2 shows the steepest slope among the three. Several scatter points cluster near 50 to 75 percent at the upper temperature range. Section b, subplot Max Month 1: The horizontal axis spans approximately 10 to 17.5 degrees. The vertical axis spans 0 to 100 percent. Month 1 fitted line rises from approximately 15 percent at 10 degrees to approximately 70 percent at 17 degrees. Month 2 and Month 3 series show progressively wider confidence intervals. Month 3 has the widest shaded band, indicating greater uncertainty. A small number of scatter points appear above 75 percent near 17 degrees, representing high-end values in the dataset.

Navigate back to Figure 4.

## Figure 5 Long description

The bar graph has two vertical bars labeled Uninfected and Infected. The horizontal axis label reads Total length left parenthesis millimetre right parenthesis. The vertical axis shows tick labels 11, 12, 13, 14, 15, 16, 17. The Uninfected bar reaches 15. A vertical error bar is drawn above and below the top of the bar. The Infected bar reaches 14. A vertical error bar is drawn above and below the top of the bar. A small animal illustration appears above each bar.

Navigate back to Figure 5.

## Table 1 Long description

The table lists repeated plankton-net samples by date, reporting the number of euphausiids examined, how many were infected, how many free-living parasite stages were collected, and summary infection metrics (prevalence, mean abundance, mean intensity). Early sampling in September to November 2023 shows mostly low to moderate prevalence, ranging from about 6 to 20 percent, with a higher value of 16 percent on 25 Oct. The highest prevalence among larger samples occurs in January 2024, reaching 24.1 percent on 4 Jan and 28 percent on 16 Jan; February includes a very large sample on 19 Feb with 973 euphausiids but a lower prevalence of 9.2 percent. From April 2024 onward, prevalence is generally low, often near 0 to 5 percent, including several zero-infection dates (30 Apr, 28 May, 23 Jul, 27 Aug). Free-living stages are usually absent or rare, but a notable spike occurs on 20 Jun 2024 with 8 free-living individuals while prevalence is only 1.1 percent. Mean intensity for infected euphausiids stays close to one to one and a half parasites per infected host across most dates, indicating limited variation in parasite load among infected individuals. Some dates have very small sample sizes, including one euphausiid on 7 Dec and two on 1 Mar, so their prevalence values are less stable and should be interpreted cautiously. Dashes indicate metrics not reported when no infections were observed or when calculations were not provided for that sample.

Navigate back to Table 1.

## Table 2 Long description

Generalized linear model results link sea surface temperature at several time windows before sampling (collection day, prior weeks and months, and prior-month maximum) to four parasite descriptors. For prevalence, temperature is a strong positive predictor from collection day through Month 1 and the prior-month maximum, with the largest deviance explained around Week 2 (about 59 percent); Month 2 weakens after correction and Month 3 is not significant. Mean abundance shows the same pattern, with strong positive effects from collection day through Month 1 and the prior-month maximum, peaking near Week 2 (about 65 percent deviance explained); Month 2 and Month 3 are not significant after correction. Mean intensity shows no meaningful association with temperature at any time window, with very low deviance explained throughout. The number of third-stage free-living individuals also shows no significant temperature relationships, with near-zero deviance explained. P-values are provided both uncorrected and Bonferroni-corrected, so only effects that remain small after correction should be treated as robust.

Navigate back to Table 2.
